# ﻿*Chaenothecopsis* (Mycocaliciales, Ascomycota) from exudates of endemic New Zealand Podocarpaceae

**DOI:** 10.3897/mycokeys.95.97601

**Published:** 2023-02-16

**Authors:** Christina Beimforde, Alexander R. Schmidt, Hanna Tuovila, Uwe Kaulfuss, Juliane Germer, William G. Lee, Jouko Rikkinen

**Affiliations:** 1 Department of Geobiology, University of Göttingen, Goldschmidtstraße 3, 37077, Göttingen, Germany; 2 Finnish Museum of Natural History, University of Helsinki, P.O. Box 7, 00014, Helsinki, Finland; 3 Johann-Friedrich-Blumenbach Institute of Zoology and Anthropology, University of Göttingen, Untere Karspüle 2, 37073, Göttingen, Germany; 4 Landcare Research, Private Bag 1930, Dunedin 9016, New Zealand; 5 School of Biological Sciences, University of Auckland, Private Bag 9209, Auckland 1142, New Zealand; 6 Organismal and Evolutionary Biology Research Programme, Faculty of Biological and Environmental Sciences, University of Helsinki, P.O. Box 65, 00014, Helsinki, Finland

**Keywords:** *Chaenothecopsis*, Mycocaliciales, New Zealand, *Phyllocladus*, plant exudate, Podocarpaceae, *Prumnopitys*, resinicolous fungi

## Abstract

The order Mycocaliciales (Ascomycota) comprises fungal species with diverse, often highly specialized substrate ecologies. Particularly within the genus *Chaenothecopsis*, many species exclusively occur on fresh and solidified resins or other exudates of vascular plants. In New Zealand, the only previously known species growing on plant exudate is *Chaenothecopsisschefflerae*, found on several endemic angiosperms in the family Araliaceae. Here we describe three new species; *Chaenothecopsismatai* Rikkinen, Beimforde, Tuovila & A.R. Schmidt, *C.nodosa* Beimforde, Tuovila, Rikkinen & A.R. Schmidt, and *C.novae-zelandiae* Rikkinen, Beimforde, Tuovila & A.R. Schmidt, all growing on exudates of endemic New Zealand conifers of the Podocarpaceae family, particularly on *Prumnopitystaxifolia*. Phylogenetic analyses based on ribosomal DNA regions (ITS and LSU) grouped them into a distinct, monophyletic clade. This, as well as the restricted host range, suggests that all three taxa are endemic to New Zealand. Copious insect frass between the ascomata contain ascospores or show an early stage of ascomata development, indicating that the fungi are spread by insects. The three new species represent the first evidence of *Chaenothecopsis* from any Podocarpaceae species and the first from any gymnosperm exudates in New Zealand.

## ﻿Introduction

The order Mycocaliciales Tibell & Wedin represents an isolated lineage of non-lichenized ascomycetes with sessile or pin-like ascomata (Tibell and Wedin 2000). Species of this lineage are currently assigned to two families and five genera of which *Chaenothecopsis* Vain. represents the largest genus. However, generic delimitations within the Mycocaliciales are in need of revision, since molecular studies show that the currently established genera are not monophyletic (e.g. Tibell and Vinuesa 2005; Tuovila 2013).

The substrate ecology of mycocalicoid species currently assigned to *Chaenothecopsis* is particularly diverse. There are many highly specialized species that have adapted to utilize specific substrates of certain tree species (Tibell 1987; Tuovila 2013) or to live in association with lichens or green algae (Titov 2006). Within *Chaenothecopsis* a number of species occur exclusively on fresh and recently solidified exudates of diverse gymnosperms and angiosperms, with most of them exhibiting a high level of host specificity (e.g. Tibell and Titov 1995; Tuovila et al. 2013). Most resinicolous *Chaenothecopsis* species are known from terpenoid conifer resins of temperate boreal forests of the Northern Hemisphere including species of *Abies* Mill., *Larix* Mill., *Picea* A.Dietr., *Pinus* L. and *Tsuga* Carrière (e.g. Titov and Tibell 1993; Tibell and Titov 1995; Rikkinen 1999, 2003; Tuovila et al. 2011b). Only two species have so far been reported from conifers of warm temperate forests in Asia (*Cunninghamia* R.Br.; Tuovila et al. 2013) and an araucarian conifer from New Caledonia (*Agathis* Salisb.; Rikkinen et al. 2014). Additional *Chaenothecopsis* species, all belonging to a distinct, monophyletic group, grow on angiosperm exudates of host trees in the Sapindales Juss. ex Bercht. & J. Presl., including Anacardiaceae R.Br. (*Khaya* A.Juss. and *Rhus* L.; Tuovila et al. 2011a) and Simaroubaceae DC. (*Ailanthus* Desf.; Tuovila et al. 2014), as well as the Apiales Nakai (*Kalopanax* Miq. (Tuovila et al. 2014), *Pseudopanax* K.Koch (Beimforde et al. 2017), and *Schefflera* J.R.Forst. & G.Forst. (Samuels and Buchanan 1983)). Of the mycocalicioid fungi so far known from New Zealand, most species of *Chaenothecopsis* are believed to be more or less cosmopolitan and live as saprophytes on the lignum of local conifers or angiosperms (Tibell 1987). Only one New Zealand species, *Chaenothecopsisschefflerae* (Samuels & D.E. Buchanan) Tibell, is known from plant exudates so far. It occurs exclusively on angiosperm exudates produced by different species of endemic Araliaceae Juss. (*Schefflera*, *Pseudopanax*; Samuels and Buchanan 1983; Beimforde et al. 2017).

Several fossils in Paleogene amber demonstrate that the ascoma morphology and resinicolous ecology of conifer-associated taxa have remained unchanged for tens of millions of years (Rikkinen and Poinar 2000; Tuovila et al. 2013; Rikkinen et al. 2018; Rikkinen and Schmidt 2018), but the evolutionary origin of the resinicolous ecology within the Mycocaliciales is still unclear. Molecular phylogenetic analyses indicate that the resinicolous ecology on conifer resin predates fungi occupying angiosperm exudate. *Chaenothecopsis* species from angiosperm exudates are grouped in a well-supported monophyletic group, suggesting a single origin of this ecological mode, whereas species on conifer resin are scattered throughout the genus, suggesting a longer evolutionary history (e.g. Rikkinen et al. 2014; Tuovila et al. 2014; Beimforde et al. 2017).

Here we describe three new *Chaenothecopsis* species that grow mainly on exudates of *Prumnopitystaxifolia* (Banks & Sol. ex D. Don) de Laub. (Podocarpaceae Endl.), an endemic New Zealand gymnosperm also known as black pine or Mataī. The morphology of each species is examined using light and scanning electron microscopy (SEM) and their phylogenetic relationships are elucidated based on ribosomal DNA data of the internal transcribed spacer region (ITS) and the large ribosomal subunit (nucLSU). The new species are described as *Chaenothecopsismatai*, *C.nodosa* and *C.novae-zelandiae*. They represent the first *Chaenothecopsis* species from any species of the conifer family Podocarpaceae and the first report of *Chaenothecopsis* species associated with gymnosperm exudate from New Zealand.

## ﻿Methods

### ﻿Biological material

**Figure 1.**
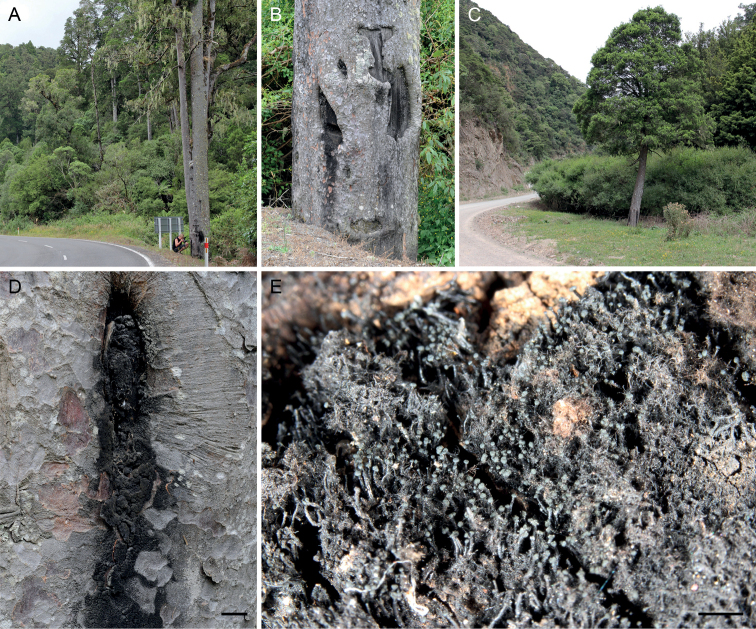
Typical habitats of *Chaenothecopsis* species from Podocarpaceae in northern New Zealand **A** collecting specimens of *Chaenothecopsisnovae-zelandiae* (PDD 110742) from a trunk of *Prumnopitystaxifolia* along Te Whaiti Road **B** (detail of **A**): *Prumnopitystaxifolia* with old, partly charred lesions **C***Prumnopitystaxifolia* hosting *Chaenothecopsismatai* (PDD 110746) along Ruatahuna Road **D** colonized exudate of *Prumnopitystaxifolia***E** (detail of **D**): exudate colonized by *Chaenothecopsismatai* (PDD 110746). Scale bars: 4 cm (**D**); 2 cm (**E**).

*Chaenothecopsis* specimens were collected from *Prumnopitystaxifolia* (Podocarpaceae) growing in different localities in the North and South Islands of New Zealand (Fig. 1, Suppl. material 1). Specimens were also collected on exudates of *Phyllocladustrichomanoides* D. Don (Podocarpaceae) from the North Island. Type specimens are deposited in the New Zealand Fungarium (PDD), Landcare Research in Auckland (Suppl. material 1).

### ﻿Light microscopy and scanning electron microscopy

**Figure 2.**
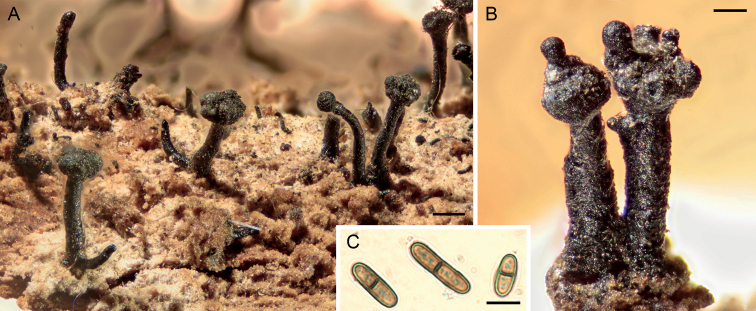
Light micrographs of *Chaenothecopsisnovae-zelandiae* sp. nov. (PDD 110744). **A** apothecia on hardened exudate of *Prumnopitystaxifolia***B** apothecia with proliferating capitula **C** ascospores. Scale bars: 200 µm (**A**); 100 µm (**B**); 5 µm (**C**).

**Figure 3.**
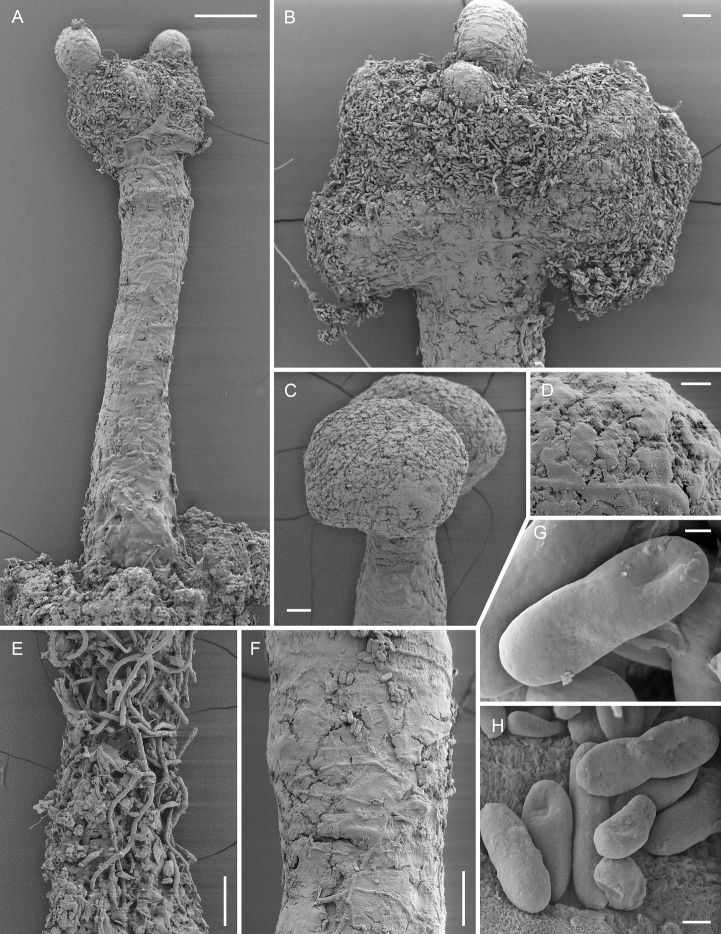
Scanning electron micrographs of *Chaenothecopsisnovae-zelandiae* sp. nov. (PDD 110744/CBNZ073B) **A** proliferating apothecium **B** mature capitulum with ascospores and amorphous material **C** semi-mature capitulum **D** (detail of **C**): epithecium of semi-mature capitulum **E** orientation of hyphae at the base of deteriorating ascoma **F** stipe surface **G** ascospore **H** ascospores. Scale bars: 100 µm (**A**); 30 µm (**B, C, E, F**); 10 µm (**D**); 2 µm (**H**); 1 µm (**G**).

**Figure 4.**
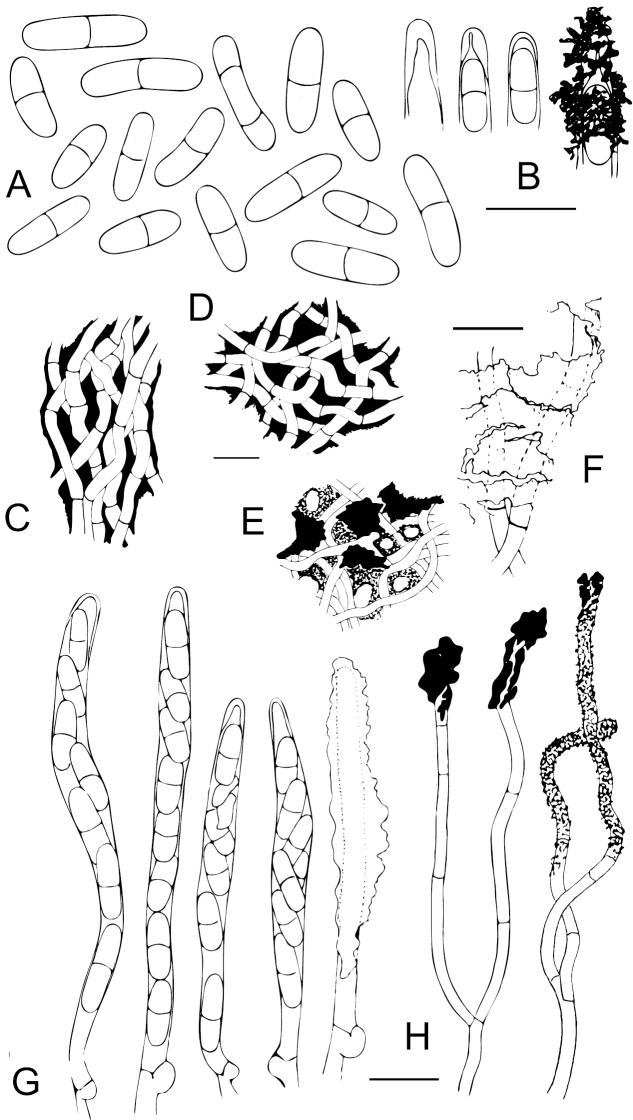
Anatomical details of *Chaenothecopsisnovae-zelandiae* sp. nov. **A** ascospores **B** ascus tips **C** excipulum **D** stipe hyphae **E** epithecium with amorphous material and pores **F** hyphae of excipulum with amorphous material **G** asci with croziers **H** paraphyses. Scale bars: 10 µm.

**Figure 5.**
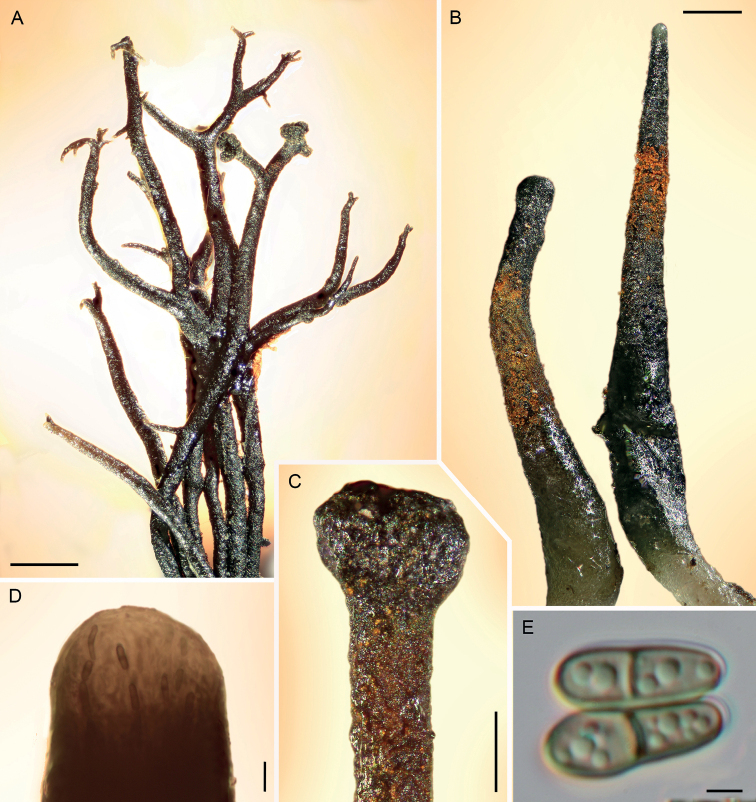
Light micrographs of *Chaenothecopsismatai* sp. nov. (PDD 110749) **A** branched and intertwined stipes, some developing capitula **B** ascomata with red pruina **C** young capitulum with ascospores **D** semi-mature capitulum **E** ascospores. Scale bars: 500 µm (**A**); 100 µm (**B, C**); 10 µm (**D**); 2 µm (**E**).

**Figure 6.**
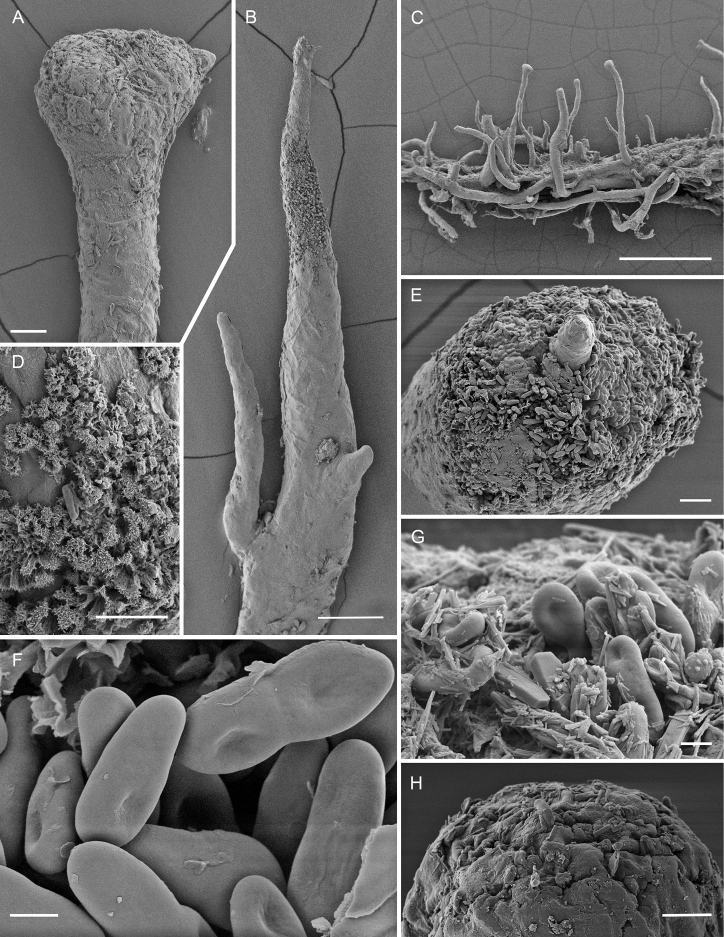
Scanning electron micrographs of *Chaenothecopsismatai* sp. nov. (PDD 110749) **A** semi-mature capitulum **B** upper part of apothecium **C** pseudostroma-like growth of apothecia **D** structure of pruina on stipe surface **E** proliferating growth of capitulum **F** ascospores **G** (detail of **E**): ascospores and crystals on capitulum surface **H** mature capitulum. Scale bars: 1 mm (**C**); 100 µm (**B**); 30 µm (**A**); 20 µm (**E**); 10 µm (**D, H**); 2 µm (**F, G**).

**Figure 7.**
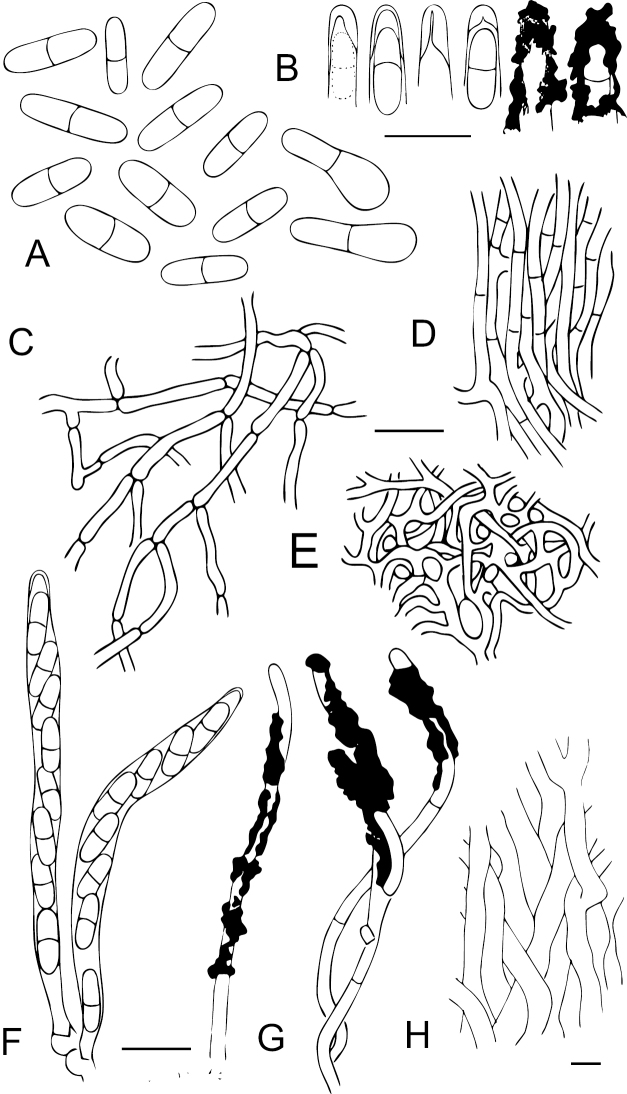
Anatomical details of *Chaenothecopsismatai* sp. nov. **A** ascospores **B** ascus tips **C** stipe hyphae **D** excipulum structure **E** epithecium structure **F** asci with corziers **G** paraphyses **H** inner stipe hyphae. Scale bars: 10 µm.

**Figure 8.**
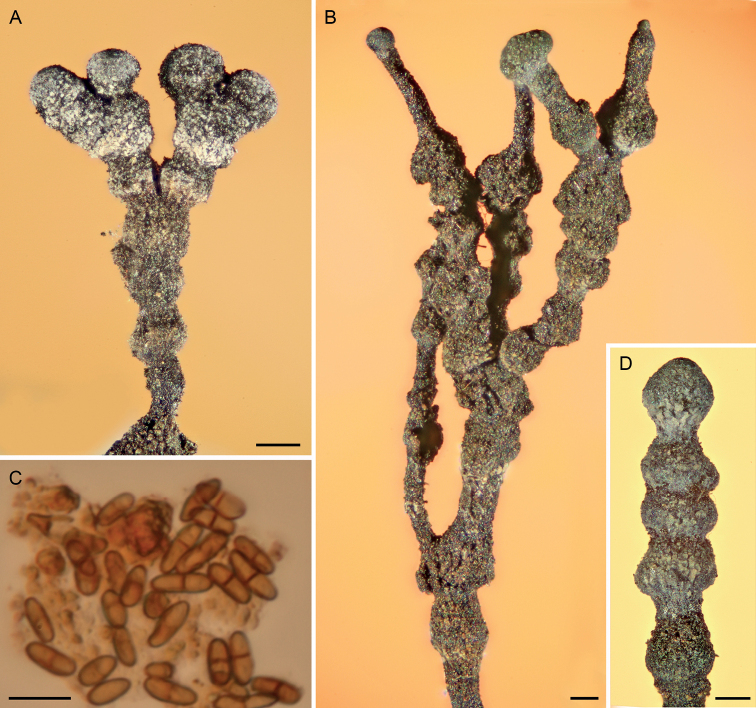
Light micrographs of *Chaenothecopsisnodosa* sp. nov. (PDD 110745) **A** branched ascoma with catenulate capitulum **B** development of this ascoma has involved at least 11 separate stages of capitulum proliferation **C** detail of compound capitulum **D** ascospores. Scale bars: 100 µm (**A, B, D**); 10 µm (**C**).

**Figure 9.**
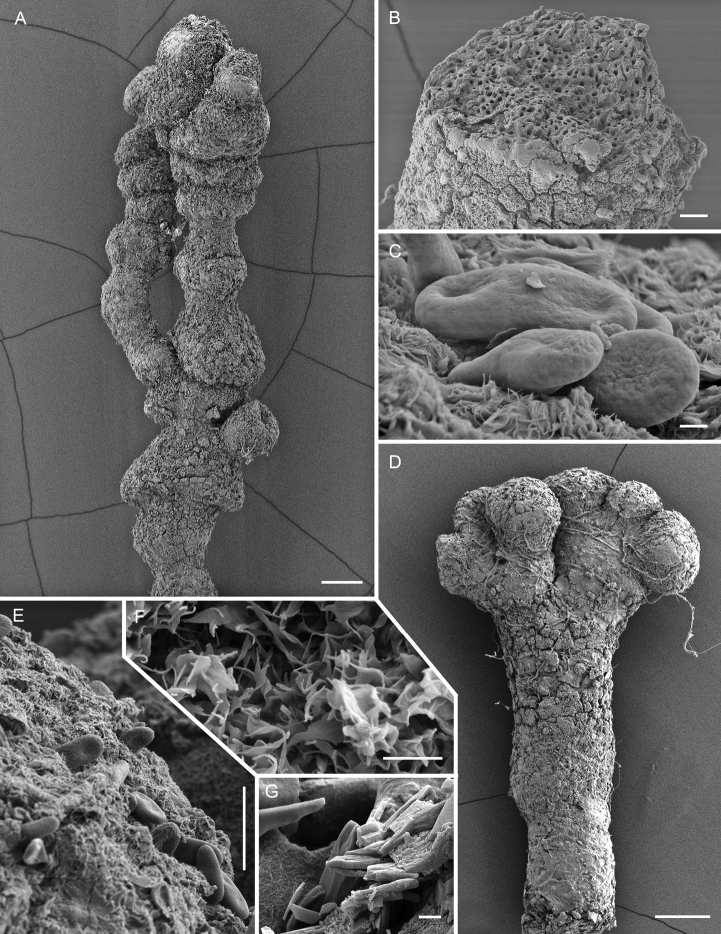
Scanning electron micrographs of *Chaenothecopsisnodosa* sp. nov. (PDD 110745) **A** branched ascoma with numerous tightly stacked capitula **B** cross section of stipe **C** ascospore ornamentation **D** compound capitula **E–G** details of capitulum surface **E** ascospores on capitulum surface **F** amorphous material on capitulum surface **G** crystals on capitulum surface. Scale bars: 100 µm (**A, D**); 10 µm (**B, E**); 1 µm (**C, F, G**).

**Figure 10.**
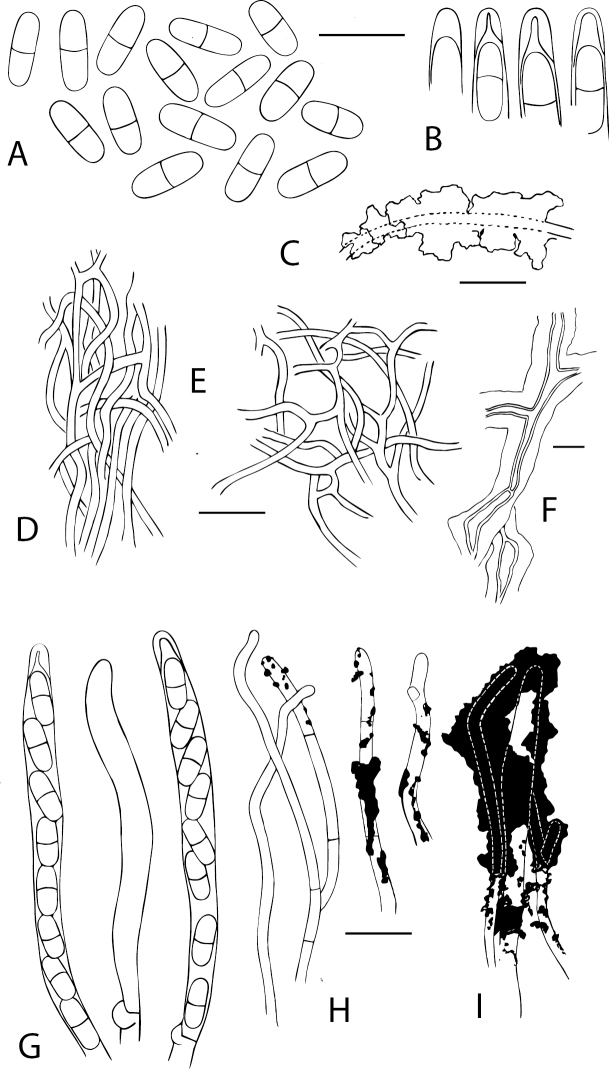
Anatomical details of *Chaenothecopsisnodosa* sp. nov. **A** ascospores **B** ascus tips **C** hypha of epithecium covered with amorphous material **D** excipulum structure **E** stipe hyphae **F** structure of the hyphae at the base of the stipe **G** asci with croziers **H** paraphyses **I** tips of paraphyses covered with amorphous material. Scale bars: 10 µm.

Morphological features (Figs 2–10) of the fungal specimens were studied and imaged using a Carl Zeiss StereoDiscovery V8 dissection microscope, a Leica DMLS microscope and a Carl Zeiss AxioScope A1 compound microscope equipped with Canon EOS 5D digital cameras. Ascomatal details were studied under 40- to 100-fold magnification, sometimes with an additional 1.6-fold magnification. Spores and inner ascomatal structures were analyzed and imaged on a microscope slide in water using Differential Interference Contrast (DIC) illumination. Some diagnostic structures, such as paraphyses and stipe hyphae, were observed by utilizing potassium hydroxide (KOH).

Light-microscopical images of ascomata on *Prumnopitys* Phil. exudates were obtained from 40–60 focal planes by using incident and transmitted light simultaneously. Individual images of focal planes were digitally stacked using the software package HeliconFocus 7.0 (Helicon Soft Limited, Kharkiv, Ukraine).

**Figure 11.**
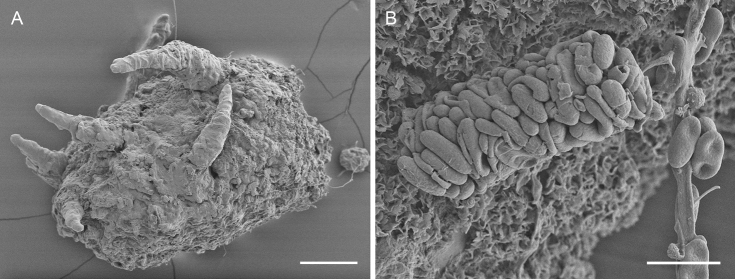
Insect fecal pellets associated with *Chaenothecopsismatai* (**A**) and *Chaenothecopsisnodosa* (**B**) **A** fecal pellet showing initial ascomata development **B** insect fecal pellets consisting predominantly of ascospores. Scale bars: 100 µm (**A**); 10 µm (**B**).

For scanning electron microscopy (Figs 3, 6, 9, 11), air dried specimens of each species were removed from the substrate, placed on a carbon-covered SEM-mount, sputtered by gold/palladium and examined under a Carl Zeiss LEO 1530 Gemini field emission scanning-electron microscope.

### ﻿Spore isolation and cultivation

Cultures were obtained by transferring single ascocarps from the substrate to cavity glass slides containing a drop of sterile 0.9% sodium chloride. All adhering substrate particles were removed and a single mature ascocarp was transferred to a fresh cavity glass slide containing a drop of sterile 0.9% sodium chloride and gently crushed with a sterile scalpel to liberate the spores. Spores were further diluted in 200–300µl sterile 0.9% sodium chloride and transferred to solid potato dextrose media (PDA, Carl Roth, Germany: 4 g/l potato infusion, 20 g/l glucose, 15 g/l agar, pH = 5.6 ± 0.2) using pipettes and filter tips. Inoculates were investigated under a Carl Zeiss StereoDiscovery V8 dissection microscope, initially every 2 days, until germination started. Cultures were subsequently stored in the dark and checked every week in order to detect possible contamination at an early stage. After 5–6 months, cultures were identified using molecular analysis of internal transcribed spacer region (ITS).

### ﻿DNA extraction, PCR amplification and sequencing

DNA was extracted from all collected representative specimens of *Chaenothecopsis*. Between 5–10 ascomata of each specimen were crushed with a fine glass mortar and pestle (Carl Roth, Karlsruhe, Germany) prior to DNA-extraction. DNA was subsequently extracted using the DNA Micro Kit from Quiagen (Hilden, Germany) following the manufacturer’s protocol, but modifying the incubation time to at least 24 hours. Samples were held in micro-glass mortars closed with parafilm during the whole incubation time.

The large subunit of nuclear ribosomal RNA (LSU) was amplified using primers pairs LR0R and LR3 (Vilgalys and Hester 1990; Rehner and Samuels 1994), as well as LR5 and LR7 (Vilgalys and Hester 1990). The internal transcribed spacer region (ITS) of the ribosomal DNA was amplified using the primers ITS5 (White et al. 1990) or ITS1F (Gardes and Bruns 1993) and ITS4 (White et al. 1990). Polymerase chain reaction (PCR) was conducted using Taq DNA polymerase (Promega, Madison, WI) by following the manufacturer’s recommendations and PCR conditions with the following steps: (1) hot start with 95 °C for 2 min; (2) 35 cycles of 45 s (ITS) to 60 s (LSU) at 95 °C, 60 s at 52–55 °C and 45 s (ITS) to 60 s (LSU) at 72 °C and (3) 10 min of final elongation at 72 °C. Subsequently, the ITS and LSU rDNA products were purified using PCRapace (Invitek, Berlin, Germany) and sequenced in both directions with a MegaBACE 1000 automated sequencing machine and DYEnamic ET Primer DNA sequencing reagent (Amersham Biosciences, Little Chalfont, UK). Sequences were assembled and edited using Bioedit 5.0.9 (Hall 1999).

### ﻿Taxon sampling and phylogenetic analysis

While many different *Chaenothecopsis* species have been reported from New Zealand (Tibell 1987), sequences of only a few, including *Chaenothecopsisdebilis* (Sm.) Tibell, *C.haematopus* Tibell and *C.schefflerae* (Samuels & D.E. Buchanan) Tibell, are available at present in Genbank. Most other sequences were obtained from specimens collected in Europe, primarily Sweden. Some Genbank sequences originating from cultures appeared inconsistent with the sequences from corresponding type material and were excluded from our analyses.

ITS and nucLSU from New Zealand specimens were sequenced in forward and backward direction and sequences were assembled using Bioedit 5.0.9 (Hall 1999). ITS and LSU data sets were aligned separately using MAFFT version 6 (Katoh and Toh 2008) and subsequently combined in Bioedit 5.0.9 (Hall 1999). For phylogenetic analyses only unambiguously alignable DNA regions were selected manually, using the mask function in Bioedit 5.0.9 (Hall 1999). The resulting data set comprises 401 basepairs (bp) of the ribosomal ITS region and 779 bp of the ribosomal LSU region.

The best fitting substitution model for each gene was chosen separately from seven substitution schemes included in the software package jModeltest 2.1.1 (Darriba et al. 2012), and models were selected according to the Bayesian information criterion (Schwarz 1978). The Bayesian information criterion supported the TIM2ef+I+G model as the best fit for the ITS region and the TrN+I+G model for the LSU gene. Both genes were combined in a single data matrix using Bioedit 5.0.9 (Hall 1999) and Bayesian analyses were carried out using Markov chain Monte Carlo in MrBayes 3.2.7 (Ronquist and Huelsenbeck 2003) on the CIPRES Science Gateway v. 3.3 (Miller et al. 2010) without using BEAGLE high-performance library (https://github.com/beagle-dev/beagle-lib).

**Figure 12.**
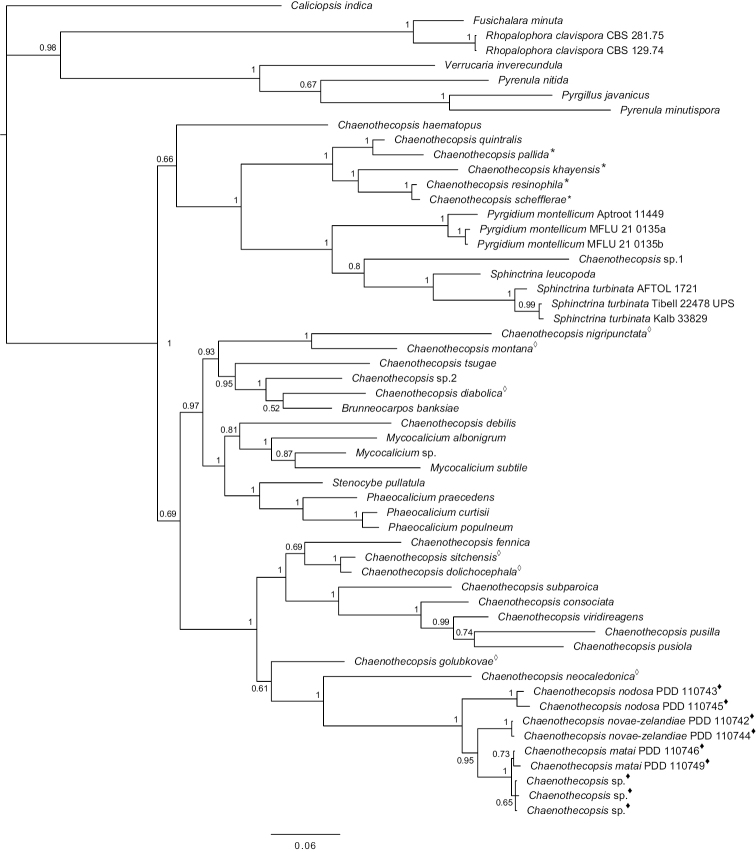
Phylogenetic relationships of mycocalicioid fungi (Mycocaliciales, Ascomycota). Bayesian tree based on partial sequences of the ribosomal internal transcribed spacer region (ITS) and the large ribosomal subunit (LSU). Numbers at branches indicate Bayesian posterior probabilities. The asterisks mark species from angiosperm exudate, white diamonds mark species from conifer resin, black diamonds mark species from podocarpous exudates.

Four chains were conducted simultaneously for 10 million generations each, sampling parameters every 1000^th^ generation. Average standard deviations of split frequency < 0.01 were interpreted as indicative of independent Markov chain Monte Carlo convergence. A burn-in sample of 2500 trees was discarded for the run and the remaining trees were used to estimate branch lengths and posterior probabilities. Convergence and sufficient chain mixing (effective sample sizes > 200) were controlled using Tracer 1.7.2 (Rambaut and Drummond 2009). GenBank accession numbers of all fungal specimens used for phylogenetic reconstruction are provided in Table 1. The combined data matrix, settings for the Bayesian analyses, and resulting phylogenetic tree (Fig. 12) were deposited in TreeBASE, direct access: http://purl.org/phylo/treebase/phylows/study/TB2:S29864.

**Table 1.** GenBank accessions for the fungal ITS and LSU sequences used in this study for phylogenetic analysis (Fig. 12).

Species name	Voucher	GenBank accessions ITS/LSU	References
*Brunneocarposbanksiae* Giraldo & Crous	CPC 29841	NR_147648/NG_066277	Crous et al. (2016)
*Caliciopsisindica* J. Pratibha & Bhat	GUFCC 4947	GQ259981/GQ259980	Pratibha et al. (2011)
*Chaenothecopsis* sp. 1	Tuovila 09-052	X119110/JX119119	Tuovila et al. (2013)
*Chaenothecopsis* sp. 2	08-004 (TUR)	KC590480/KC590485	Tuovila (2014)
*Chaenothecopsisconsociata* (Nádv.) A.F.W. Schmidt	Tibell 22472 (UPS)	AY795851/DQ008999	Tibell and Vinuesa (2005)
*Chaenothecopsisdebilis* (Sm.) Tibell	Tibell 16643 (UPS)	AY795852/ AY795991	Tibell and Vinuesa (2005)
*Chaenothecopsisdiabolica* Rikkinen & Tuovila	H:Tuovila 06-035	JX119109/JX119114	Tuovila (2013)
*Chaenothecopsisdolichocephala* Titov	Tibell 19281	AY795854/AY795993	Tibell and Vinuesa (2005)
*Chaenothecopsisfennica* (Laurila) Tibell	Tibell 16024 (UPS)	AY795857/AY795995	Tibell and Vinuesa (2005)
*Chaenothecopsisgolubkovae* Tibell & Titov	Titov 6707 (UPS)	AY795859/AY795996	Tibell and Vinuesa (2005)
*Chaenothecopsishaematopus* Tibell	16625 (UPS)	AY795861/AY795997	Tibell and Vinuesa (2005)
*Chaenothecopsiskhayensis* Rikkinen & Tuovila	JR 04G058	JX122785/HQ172895	Tuovila et al. (2011a)
*Chaenothecopsismontana* Rikkinen	H:Tuovila 07-086	JX119105/JX119114	Tuovila et al. (2013)
*Chaenothecopsisneocaledonica* Rikkinen, Tuovila & A.R. Schmidt	Rikkinen 010179	KF815196/KF815197	Rikkinen et al. (2014)
*Chaenothecopsisnigripunctata* Rikkinen	H:Tuovila 06-013	JX119103/JX119112	Tuovila et al. (2013)
*Chaenothecopsismatai* Rikkinen, Beimforde, Tuovila & A.R. Schmidt	PDD 110746	OQ308931/OQ308874	This study
	PDD 110749	OQ308932/OQ308875	This study
*Chaenothecopsisnodosa* Beimforde, Tuovila, Rikkinen & A.R. Schmidt	PDD 110743	OQ308933/OQ308876	This study
	PDD 110745	OQ308934/OQ308877	This study
*Chaenothecopsisnovae-zelandiae* Rikkinen, Beimforde, Tuovila & A.R. Schmidt	PDD 110742	OQ308935/OQ308878	This study
	PDD 110744	OQ308936/OQ308879	This study
*Chaenothecopsispallida* Rikkinen & Tuovila	H:JR 010652	JX122779/JX122781	Tuovila et al. (2013)
*Chaenothecopsispusilla* (A. Massal.) A.F.W. Schmidt	Tibell 16580 (UPS)	-/ DQ009000.1	Tibell and Vinuesa (2005)
*Chaenothecopsispusiola* (Ach.) Vain.	H:Tuovila 09-047	JX119106/JX119115	Tuovila et al. (2013)
*Chaenothecopsisquintralis* Messuti, Amico, Lorenzo & Vidal-Russ.	BCRU:05233	-/JQ267741	Messuti et al. (2012)
*Chaenothecopsisresinophila* Rikkinen & Tuovila	H:JR000424	JX122780/JX122782	Tuovila et al. (2013)
*Chaenothecopsisschefflerae* (Samuels & D.E. Buchanan) Tibell	Rikkinen 13183	KY499965/ KY499967	Beimforde et al. (2017)
*Chaenothecopsissitchensis* Rikkinen	H:Tuovila 06-033	JX119102/JX119111	Tuovila et al. (2013)
*Chaenothecopsissubparoica* (Nyl.) Tibell	Tretiach (hb. Tretiach)	AY795869/-	Tibell and Vinuesa (2005)
*Chaenothecopsistsugae*	H:JR07005B	JX119104/JX119113	Tuovila et al. (2013)
*Chaenothecopsisviridireagens* Rikkinen	Tibell 22803 (UPS)	AY795872/ DQ013257	Tibell and Vinuesa (2005)
*Fusichalaraminuta* Hol.-Jech.	CBS 709.88	KX537754/ KX537758	Réblová et al. (2017)
*Mycocaliciumalbonigrum* (Nyl.) Tibell	Tibell 19038	AF223966/ AY796001	Tibell and Vinuesa (2005)
*Mycocaliciumsubtile* (Pers.) Szatala	JR6450	OQ308930/OQ308873	This study
*Mycocalicium* sp.	Tuovila 09-131 (TUR)	KC590482/KC590487	Tuovila et al. (2014)
*Sphinctrinaleucopoda* Nyl.	Kalb 33829 (hb. Kalb)	AY795875/AY796006	Tibell and Vinuesa (2005)
*Sphinctrinaturbinata* (Pers.) De Not.	Tibell 23093 (UPS)	AY795877/DQ009001	Tibell and Vinuesa (2005)
	Tibell 22478 (UPS)	AY795876/-	Geiser et al. (2006)
	AFTOL-ID 1721	-/ EF413632	Geiser et al. (2006)
*Stenocybepullatula* (Ach.) Stein	Tibell 17117 (UPS)	AY795878/AY796008	Tibell and Vinuesa (2005)
*Phaeocaliciumpopulneum* (Brond. & Duby) A.F.W. Schmidt	Tibell 19286 (UPS)	AY795874/AY796009	Tibell and Vinuesa (2005)
*Phaeocaliciumpraecedens* (Nyl.) A.F.W. Schmidt	Tuovila 09-240 (TUR)	KC590481/KC590486	Tuovila et al. (2014)
*Pyrgillusjavanicus* (Mont. & Bosch) Nyl.	AFTOL-ID 342	DQ826741/DQ823103	James et al. (2006)
*Pyrenulaminutispora* Aptroot & M. Cáceres	ABL AA11877	KT820119/-	Gueidan et al. (2016)
*Pyrenulanitida* (Weigel) Ach.	F 5929	JQ927458/ DQ329023	del Prado et al. (2006); Weerakoon et al. (2016)
*Rhopalophoraclavispora* (W. Gams) Réblová	CBS 129.74	KX537751/ MH872573	Réblová et al. (2017)
	CBS 281.75	KX537752/ KX537756	Réblová et al. (2017)
*Verrucariainverecundula* Pykälä & Myllys	FILIC650-13	MK138796/-	Pykälä et al. (2019)

## ﻿Results

### ﻿Taxonomy

#### ﻿ Chaenothecopsis novae-zelandiae

Taxon classificationFungiMycocalicialesMycocaliciaceae

Rikkinen, Beimforde, Tuovila & A.R. Schmidt sp. nov.

213FA4AB-516F-5AAF-98AB-B238F92C2C29

MB846458

Figs 2 , 3 , 4

##### Type.

New Zealand, South Island, State Highway 6 close to Makarora, Otago, ca. 44°13.787'S, 169°13.9708'E, on exudate of *Prumnopitystaxifolia*, 5 February 2017, holotype: PDD110744, New Zealand Fungarium (PDD), Landcare Research in Auckland, GenBank accession OQ308936/OQ308879.

##### Diagnosis.

*Chaenothecopsisnovae-zelandiae* differs from other *Chaenothecopsis* species by forming mostly solitary ascomata on podocarpous plant exudates, and by having inner ascomatal structures firmly connected by amorphous material and finely ornamented spores, which can be slightly constricted at the septum.

##### Etymology.

The specific epithet refers to New Zealand where the species was first discovered.

##### Description.

***Apothecia*** growing on the exudate of *Prumnopitystaxifolia*, 0.6–1.6 mm tall, growing individually or grouped in small clusters, often branched or proliferating from the capitulum. ***Stipe*** glossy black, straight, 80–180 µm wide, sometimes slightly flexuous or curved, frequently branched at the base or, more rarely, in the upper parts. ***Stipe hyphae*** mostly covered with a layer of hard pigment partly dissolving in KOH, 6–8 µm wide, with walls two layered, the outer wall brown, 2–4 µm wide and cell walls fused, the inner wall pale to hyaline, *c.* 0.5–1.5 µm wide, with the hyphae intertwined (textura intricata prismatica), swelling in KOH and the yellowish brown pigment leaking into the medium; hyphae in inner part of the stipe hyaline, slightly intertwined, 3–4.6 µm, swelling in KOH. ***Capitulum*** black, in young apothecia hemispherical to sometimes almost spherical, sometimes lobed or multi-headed, 200–400 µm wide. ***Excipulum*** hyphae brownish to slightly green, 5–7 µm wide, periclinally arranged or slightly intertwined (textura prismatica), swelling in KOH, with some brown pigment leaking into the medium; wall 2–2.5 µm. ***Epithecium*** light green to emerald green, appearing as a crustose layer, usually with crystals, composed of hyphae extending from the excipulum; hyphae attached to the hymenium by the amorphous material; containing various amounts of orange to ruby-red pigment in most ascomata, usually occurring as crystals on the outer walls of hyphae, and sometimes also inside their lumina. ***Hypothecium*** light green to hyaline, with the hyphae swelling in KOH. ***Hymenium*** light brown to greenish to almost hyaline, swelling in KOH, full of amorphous material strongly congealing the asci and paraphyses together. ***Paraphyses*** hyaline, filiform, 1.5–2 µm wide (n = 10), branched, as long or slightly longer than the asci, variously covered with amorphous material, septate at 10–15 µm intervals. ***Asci*** cylindrical, 55–60 × 6.1 µm (n = 5), with the apex variously thickened, often penetrated by a short canal; mature asci usually without a thickening, variously covered with light green to hyaline, amorphous material, formed with croziers. ***Ascospores*** uniseriate, sometimes partly biseriate, obliquely to periclinally oriented in asci, 1-septate, light brown, cylindrical to slightly ellipsoid, sometimes phaseoliform, smooth, or with a very fine ornamentation, (7.7–) 8–13 (–15.4) × (2.8–) 3–3.9 (–4.5) µm (n = 70) [mean 10.3 × 3.4 µm, Q = (2.1–) 2.4–3.8 (–5.0), mean Q = 3.1]; septa as thick as the spore wall, sometimes constricted.

##### Ecology and distribution.

*Chaenothecopsisnovae-zelandiae* has been found only at two locations in temperate broad-leaved rainforests of New Zealand on semi-hardened exudate and exudate-soaked bark on the main trunk of *Prumnopitystaxifolia*, sometimes growing mixed with *Chaenothecopsismatai*.

##### Specimens examined.

Specimens PDD110744 (Figs 2, 3A, B, F–H) and PDD 110742 (Figs 1A, B, 3C, D, E) on exudate of *Prumnopitystaxifolia*. The specimens are deposited in the New Zealand Fungarium (PDD), Landcare Research in Auckland, with a duplicate specimen (PDD 110742/JR13033) in Helsinki (H). The collection data and GenBank accession numbers are given in Suppl. material 1.

#### ﻿ Chaenothecopsis matai

Taxon classificationFungiMycocalicialesMycocaliciaceae

Rikkinen, Beimforde, Tuovila & A.R. Schmidt sp. nov.

90F17D37-7293-5097-BF4F-0D9275607EF9

MB846459

Figs 5 , 6 , 7

##### Type.

New Zealand, South Island, Croydon Bush, Dolamore Park, Southland, ca. 46°3.6657'S, 168°49.9135'E, on exudate of *Prumnopitystaxifolia*. 17 February 2017, Beimforde PDD110749, holotype; New Zealand Fungarium (PDD), Landcare Research in Auckland, GenBank accession OQ308932/OQ308875.

##### Diagnosis.

*Chaenothecopsismatai* differs from other *Chaenothecopsis* species by forming extensive mat-like pseudostromata on podocarpous plant exudates with long, often multi-branched, partially translucent stipes, predominantly slender capitula and smooth septate spores that are often constricted at the septum.

##### Etymology.

The specific epithet refers to the Maori name of *Prumnopitystaxifolia*, the exudate-producing tree on which the species was first discovered.

##### Description.

***Apothecia*** growing on the exudate of *Prumnopitystaxifolia*, arising from a dense mycelium mat which hardens in dry conditions and swells under humid conditions, forming a loose intertwined network with apices either remaining sterile or developing capitula, sometimes growing individually. ***Stipe*** glossy, crustose near stipe apices and pruinose parts, black to brownish, often with a hyaline base and/or apex, 90–240 μm wide, usually 2–7 mm long, or sometimes more than 1 cm long, flexuous or curved, multiple-branched, mostly uniformly thickened, tapering towards the apices, often with an orange to red pruina below the capitula. ***Stipe hyphae*** 2–8 µm wide, with walls two-layered, the outer wall brown and the cell walls fused, the inner walls hyaline, *c.* 0.5–1 µm wide, with the hyphae intertwined (textura prismatica-intricata), swelling in KOH; hyphae in the inner part of stipe hyaline to greenish, 2–6 µm wide, swelling in KOH. ***Capitulum*** black, 110–220 µm wide, 100–200 high, lentiform to cupulate, sometimes narrower than or as wide as the stipe. ***Excipulum*** hyphae brown to emerald green, 4–7 µm wide, intertwined (textura prismatica-intricata), with outer cell walls fused, swelling in KOH and some brown pigment leaking into the medium. ***Epithecium*** brownish to emerald green to hyaline, appearing as crusty layer, usually with crystals, composed of the hyphae of the excipulum and paraphyses forming a variously thickened layer. Containing various amounts of orange to ruby-red pigments in most ascomata, usually occurring as crystals on the outer walls of hyphae, and sometimes also inside their lumina. ***Hypothecium*** light brown to greenish hyaline, with the hyphae swelling in KOH. ***Hymenium*** brownish to emerald to hyaline, with the hyphae swelling in KOH, orange to red pigments present, full of amorphous material strongly congealing asci and paraphyes together. ***Paraphyses*** hyaline, filiform, 1.5–2 µm wide (n = 10), branched, usually slightly longer than the asci, variously covered with amorphous material, septate at 9–19 µm intervals. ***Asci*** cylindrical, 47–77 µm high, 5–7 µm wide (n = 8), with the apex variously thickened, often penetrated by a poorly developed canal; mature asci usually without a thickening, formed with croziers, tightly embedded in the hymenium, with light brown-green to hyaline amorphous material making individual asci difficult to observe. ***Ascospores***, smooth, uniseriate, periclinally (to slightly obliquely) oriented in asci, 1-septate, brown, cylindrical to slightly ellipsoid, (7.3–) 8–12.5 (–14) × (2.8–) 3–4.5 (–4.7) µm (n = 60), [mean 10.3 × 3.4 µm, Q = (2–) 3–4.3 (–4.5), mean Q = 3.2]; septa as thick as spore wall, sometimes constricted.

##### Ecology and distribution.

*Chaenothecopsismatai* has been found at several locations in temperate broad-leaved rain forests of New Zealand on semi-hardened exudate and exudate-soaked wood and bark on the main trunk of *Prumnopitystaxifolia*, sometimes growing mixed with *Chaenothecopsisnovae-zelandiae*. Some specimens of a morphologically-similar *Chaentohecopsis* species have also been collected from exudate of *Phyllocladustrichomanoides* (Podocarpaceae), but their detailed analysis awaits more material.

##### Specimens examined.

PDD110746 (Fig. 1D–E), PDD110747, PDD110748, PDD110749 (Figs 5, 6) on exudate of *Prumnopitystaxifolia*. The specimens are deposited in the New Zealand Fungarium (PDD), Landcare Research, Auckland, with a duplicate of specimen JR13032 in Helsinki (H). The collection data and GenBank accession numbers are given in Suppl. material 1.

#### ﻿ Chaenothecopsis nodosa

Taxon classificationFungiMycocalicialesMycocaliciaceae

Beimforde, Tuovila, Rikkinen & A.R. Schmidt sp. nov.

8018A732-9725-5BC3-87EA-748B8F380C76

MB846460

Figs 8 , 9 , 10

##### Type.

New Zealand, North Island, close to Kakaho Camp site, central North Island, ca. 38°34.0224'S, 175°43.0525'E, on exudate of *Prumnopitystaxifolia*, 5 April 2015, Beimforde PDD 110745, holotype; New Zealand Fungarium (PDD), Landcare Research in Auckland, GenBank accession OQ308934/OQ308877.

##### Diagnosis.

*Chaenothecopsisnodosa* differs from other *Chaenothecopsis* species by producing capitula in a catenulate stack, consecutively on top of each other, typically covered with a white pruina.

##### Etymology.

The specific epithet refers to the appearance of catenulate groups of sphaeric capitula stacked on top of each other

##### Description.

***Apothecia*** growing on the exudate of *Prumnopitystaxifolia*, 1.0–3.1 mm tall, growing individually and proliferating from the capitulum, often several from a single capitulum or from the stipe, eventually forming catenulate stacks of several capitula on top of each other. ***Stipe*** dark brown to black, straight to slightly curved, 100–190 μm wide, becoming crustose with age, often with a white pruina at upper stipe regions, and sometimes with an additional red pruina below. ***Stipe hyphae*** 3–8 µm wide, with walls two layered, the outer wall dark brown, 1.5–3.5 µm and with cell walls fused in most parts, the inner wall *c.* 0.5–1 µm, with the hyphae intertwined (textura prismatica-intricata), swelling in KOH; hyphae in inner parts yellowish to light brown, 2–5 µm wide, swelling in KOH. ***Capitulum*** black, lenticular to almost spherical or ellipsoid, 150–420 μm wide, 250–220µm high; typically a white pruina is macroscopically visible on the capitula. ***Excipulum*** hyphae light brown to hyaline in younger ascomata, brown in older ascomata, 2–6 µm wide, intertwined (textura prismatica-intricata), swelling in KOH; often covered with a crusty layer of amorphous material and crystals. ***Epithecium*** light green to moss green, appearing as a crusty layer, variously (up to 20 µm) thickened, usually with crystals, composed of hyphae extending from the excipulum; hyphae attached to the hymenium by the amorphous material. ***Hymenium*** light brown to olive green, with the hyphae swelling in KOH, full of amorphous material strongly congealing the asci and paraphyses together. ***Paraphyses*** hyaline, filiform, 1.5–2.5 μm wide (n = 20), sometimes branched, as long as or slightly longer than asci, variously covered with amorphous material, septate at 10–25 μm intervals, with the apices intertwined and agglutinated with the hyphae of the epithecium. ***Asci*** cylindrical, 60–77 × 4.9–7.7 μm (n = 8), with the apex variously thickened, penetrated by a minute canal visible only in young asci; mature asci usually without a thickening, variously covered with light green to hyaline, amorphous material, formed with croziers; asci in older capitula disintegrated. ***Ascospores*** uniseriate, obliquely to periclinally oriented in the asci, 1-septate, brown, cylindrical to slightly ellipsoid, ornamented, (6.7–) 8.5–9.2 (–10.8) × (3.1–) 3.4–3.9 (–4.6) μm (n = 60) [mean 9.5 × 3.8 μm, Q = (2.8–) 3.5–4.6 (–5.4), mean Q = 3.8]; septa as thick as spore wall.

##### Ecology and distribution.

*Chaenothecopsisnodosa* has to date been found only in temperate broad-leaved rainforests of New Zealand on semi-hardened exudate and exudate-soaked exposed wood and bark on the main trunk of *Prumnopitystaxifolia*.

##### Specimens examined.

Specimens PDD 110743 and PDD 110745 (Figs 8, 9) on exudate of *Prumnopitystaxifolia*. The specimens are deposited in the New Zealand Fungarium (PDD), Landcare Research, Auckland. The collection data and GenBank accession numbers are given in Suppl. material 1.

## ﻿Discussion

### ﻿Taxonomy and systematics

The new species described here represent the first *Chaenothecopsis* species from exudates of New Zealand gymnosperms. Only *Chaenothecopsisschefflerae* had previously been found on New Zealand plant exudates, but this species is restricted to angiosperm exudates of endemic Araliaceae (Beimforde et al. 2017).

All three new species occur on the same substrate, i.e., exudate of *Prumnopitystaxifolia* and each has a distinctive macroscopic appearance. *Chaenothecopsisnodosa* tends to produce many capitula in a catenulate stack, consecutively on top of each other (Figs 8A, B, D, 9A) and typically produces a white prunia (Fig. 8A, D). In contrast, *C.matai* and *C.novae-zelandiae* produce a reddish pruina (Fig. 5B, C). Ascomata of *C.novae-zelandiae* have comparatively short stipes and tend to grow individually or in smaller groups (Fig. 2A), whereas *C.matai* usually produces extensive mat-like pseudostromata on its substrate (Figs 5A, 6C).

*Chaenothecopsismatai* may form very long, multiply-branched and interwoven stipes, often with hyaline parts at the base or apex (Fig. 5B). This species grows in areas of the host trees where exudate accumulates in a humid environment, e.g., in crevices of trunks or branches, or between forking trunks at the base of trees. In such places, *C.matai* sometimes forms dense mycelial mats which are soaked with the water-soluble *Prumnopitys* exudate and from which apothecia and sterile stalks arise, forming a pseudostroma-like network. A pseudostroma-like growth habit has also been observed in *Chaenothecopsiscaespitosa* (W. Phillips) D. Hawksw., described by Hawksworth (1980). However, in contrast to *C.matai*, apothecia of *C.caespitosa* grow in tuft-like structures. Nor does *C.caespitosa* produce the long, abundantly branched stipes observed in *C.matai*. In addition, the former species has only been collected from rotting polypores on *Taxus* branches in Great Britain. A pseudostroma-like growth habit is also known from *Mycocaliciumsequoia* Bonar (Bonar 1971), a mycocalicioid species growing on exudates of *Sequoia* Endl. and *Sequoiadendron* J.Buchholz. However, in contrast to *C.matai*, *M.sequioae* has a bright yellow pruina on the capitulum surface and tends to produce very compact stroma-like mycelia in which the stalked ascomata are almost completely embedded.

*Chaenothecopsisnodosa* is morphologically conspicuous and readily distinguishable from *C.matai*, *C.novae-zelandiae* and other resinicolous *Chaenothecopsis* species with proliferating ascomata, such as *C.diabolica* Rikkinen & Tuovila (Tuovila et al. 2011b), *C.dolichocephala* Titov (Tibell and Titov 1995), and *C.proliferatus* Rikkinen, A. R. Schmidt & Tuovila (Tuovila et al. 2013) on the basis of its catenulate, very tightly stacked capitula. Proliferating ascomata are produced by several resinicolous *Chaenothecopsis* species from different clades, and are also evident from fossil specimens from Paleogene Baltic and Bitterfeld amber (Tuovila et al. 2013; Rikkinen et al. 2018). One can assume that these types of ascomata can effectively rejuvenate if partially overrun by fresh exudate and thus represent a morphological adaptation to life on plant exudates (Tuovila et al. 2013).

In Mycocaliciales, the assignment of species to particular genera, and the delimitation of species is sometimes challenging when using morphological characters only (Schmidt 1970; Tibell 1984, 1987; Titov 2006; Tuovila 2013). For this reason, besides careful examination of microscopical diagnostic characters (for details see Tuovila and Huhtinen 2020), we used additional information from phylogenetically informative gene regions, the internal transcribed spacer region (ITS) and the large ribosomal subunit (LSU), for species identification and taxonomic assignment. Our phylogenetic tree (Fig. 12) accentuates unresolved issues of generic delimitation within Mycocaliciales (e.g. Tibell and Vinuesa 2005; Tuovila 2013) since species assigned to genera such as *Mycocalicium* Vain., *Phaeocalicium* A.F.W. Schmidt and *Chaenothecopsis* appear not to be monophyletic. The recently erected genus *Brunneocarpos* Giraldo & Crous (Crous et al. 2016) is nested within *Chaenothecopsis*, with *C.diabolica* constituting the sister taxon of *Brunneocarposbanksiae* Giraldo & Crous.

Our phylogenetic analysis (Fig. 12) places all three new *Chaenothecopsis* species in a monophyletic clade. The three species also share many morphological features. Additional specimens collected from *Phyllocladustrichomanoides* are most similar to *C.matai*, differing only by few base pairs in the ITS region. However, due to the very limited sample material from *Phyllocladus* Rich. exudates, we were currently not able to study possible differences between *C.matai* specimens collected from *Prumnopitys* and *Phyllocladus* exudates in detail.

*Chaenothecopsisneocaledonica* Rikkinen, A.R.Schmidt & Tuovila is the sister taxon to the New Zealand clade in our phylogenetic tree (Fig. 12). *C.neocaledonica* grows on resinous plant exudates of *Agathisovata* (C.Moore ex Vieill.) Warb. (Araucariaceae Henkel & W.Hochst.), an endemic New Caledonian conifer (Rikkinen et al. 2014). This sister taxon relationship is conceivable due to their geographical proximity. Morphologically, all three New Zealand species differ from *C.neocaledonica* (and from other resinicolous species with one-septate spores) in the presence of peculiar amorphous material covering the asci and paraphyses, sometimes in a very thick layer (Figs 4B, F, H, 7B, G, 10C, H, I). This material also glues the whole hymenium tightly together and makes asci and paraphyses difficult to observe. In addition, the spores of the New Zealand species are on average narrower than those of *C.neocaledonica*, and at least some in each studied ascoma were phaseoliforme (resembling kidney-beans) or slightly constricted (*C.matai* and *C.novae-zelandiae*) at the septum, in contrast to the strictly cylindrical-fusoid spores of *C.neocaledonica*.

### ﻿Endemism and spore dispersal

Most previously known *Chaenothecopsis* species from temperate forest systems of New Zealand are considered to be cosmopolitan and not strictly host specific. According to Tibell (1987), *C.debilis*, *C.nana* Tibell, *C.nivea* (F. Wilson) Tibell, *C.pusilla* (A. Massal.) A.F.W. Schmidt and *C.savonica* (Räsänen) Tibell occur on hard lignum and/or bark of various New Zealand gymnosperms or angiosperms. Other species, such as *C.haematopus*, *C.lignicola* (Nádv.) A.F.W. Schmidt, *C.nigra* Tibell and *C.nigropedata* Tibell, may also be associated with lichens or algae.

Previously only two *Chaenothecopsis* species, *C.brevipes* Tibell and *C.schefflerae*, were thought to be endemic to New Zealand (Tibell 1987). *C.brevipes* is a lichenicolous species, characterized by its short stalk and strict association with lichens of the genus *Arthonia* Ach. (Arthoniaceae). However, this species seems to be more widespread than previously assumed. In New Zealand *C.brevipes* occurs on *Arthoniaplatygraphella* Nyl. (Tibell 1987) but was later also noted on other *Arthonia* species e.g., in Russia (Titov and Tibell 1993), North America and Canada (Selva 2010). *C.schefflerae* is a species which appears to be endemic to New Zealand as it only occurs on exudates of endemic Araliaceae. This species was initially known only from exudates of *Scheffleradigitata* (Araliaceae) but was later also found on exudates of *Pseudopanax* (Beimforde et al. 2017). In any case, *C.schefflerae* is not closely related to the species described here, as it belongs to a well-supported monophyletic group that includes all other known *Chaenothecopsis* species from angiosperm exudates.

*Chaenothecopsisnovae-zelandiae*, *C.matai* and *C.nodosa* were predominantly found on exudates of *Prumnopitystaxifolia*. However, as mentioned above, we also found very limited material of a similar *Chaenothecopsis* species growing on exudates of *Phyllocladustrichomanoides*. Thus, it is possible that the new species may also occur on exudates of other *Phyllocladus* species and possibly even on *Prumnopitysferruginea*, all of which are also endemic to New Zealand. Although a broader host range is thus possible, we expect that the three new *Chaenothecopsis* species described here all belong to New Zealand’s endemic mycobiota, both due to their specialized substrates and the fact that they group into a distinct monophyletic lineage in our phylogenetic analyses (Fig. 12).

The exudate outpourings of *Prumnopitystaxifolia* are sometimes densely covered by numerous *Chaenothecopsis* ascomata providing shelter to diverse arthropods. Some of our collected specimens, particularly those with numerous ascomata were abundantly littered with insect fecal pellets between or at the base of the ascomata. Scanning electron micrographs revealed spores on the outer surfaces of many fecal pellets, and some smaller fecal pellets consist almost entirely of *Chaenothecopsis* spores (Fig. 11B), suggesting that associated insects feed on the ascomata and defecate undigested ascospores. This notion is substantiated by our findings of fecal pellets with associated early stages of ascomata development (Fig. 11A). We detected a range of insects and insect remnants between the densely arranged ascomata in several samples, for example lepidopteran cocoons, mites, coleopterans such as a rove beetle (Staphylinidae Latreille) and possibly wood boring beetles as well as insect exuviae, pupae and larvae. These findings, together with the spores and initial ascomata development in the fecal pellets, indicate that the densely growing ascomata provide shelter and food source for diverse insects and that ascospores of the fungi are ingested, but probably not digested by insects. It is thus likely that insects are involved in the spore dispersal of the species described herein, as spores may be consumed by the insects and spread with their excrements or get attached to the insects’ surface when they crawl over the apothecia. It might well be that the spore-dispersing insects are also associated with the host trees and thus guarantee that the spores reach the substrates that are essential for the fungal species to survive.

### ﻿Ecology on plant exudates and evolution

Some fungi have developed defenses against the toxic components of plant exudates (e.g. Rautio et al. 2012; Adams et al. 2013) but it is uncertain whether this unusual, inherently toxic substrate is preferred to evade competition or whether exudates provide a nutrient source for the fungi. The dependence of some mycocalicioid fungi and other resinicolous ascomycetes on conifer resins and other plant exudates, and the fact that their hyphae grow randomly into this substrate (Beimforde et al. 2020) suggests a nutrient uptake from the exudates. Theoretically, resin and other plant exudates represent oxidizable organic matter, but it has not yet been proven empirically whether fungi are able to metabolize compounds of plant exudates.

Our culture experiments demonstrate that all three species described here grow *in vitro* on a carbohydrate-based medium (PDA). Still, we cannot exclude that phenolic and/or terpenoid substances of the *Prumnopitys* exudate may also be degraded by the species. The composition of plant exudate differs greatly between individual plant lineages. The exudates of angiosperms that serve as hosts for some *Chaenothecopsis* species (*Khaya* and *Rhus* (Anacardiaceae), *Ailanthus* (Simaroubaceae), *Kalopanax*, *Pseudopanax* and *Schefflera* (Araliaceae)) consist of complex hydrophilic, non-polymerized polysaccharides (Langenheim 2003), representing a conceivable nutrient source. In contrast, conifer host trees produce resinous exudates that consist of a mixture of hydrophobic, phenolic and terpenoid components that are toxic for most microorganisms (Bednarek and Osbourn 2009; Sipponen and Laitinen 2011; Rautio et al. 2012) because they damage cell wall structures (Rautio et al. 2011). Nevertheless, terpenoid/phenolic conifer exudates may contain hybrid subgroups such as guaiac gums, guaiac resins, and kino resins (Lambert et al. 2021), which might be degradable by fungi. The composition of *Prumnopitys* exudate has not yet been studied in detail, but it appears to differ from other conifer exudates (Lambert et al. 2007). According to our observations, the exudate of *Prumnopitystaxifolia* differs from resins or exudates of most other conifer hosts in being water-soluble, in its dark tint and the strong phenolic fragrance of fresh outpourings. This means that, as recently shown for some Araucaria species (Seyfullah et al. 2022), distinct types of exudate (gum, resin, and gum resin) may co-occur in *Prumnopitys*.

Our phylogenetic analysis indicates that the three species from Podocarpaceae exudate descend from a common ancestor. Likewise, all known *Chaenothecopsis* species from various angiosperm exudates also originate from a common ancestor. In contrast, resinicolous species from terpenoid conifer resins have multiple origins and occur in several lineages within the Mycocaliciales, suggesting a longer and more complex evolutionary history. The age of the resinicolous ecology within Mycocaliciales remains uncertain since relationships between individual monophyletic clades have not yet been fully resolved. In any case, resinicolous *Chaenothecopsis* species from various ambers prove that this ecological mode on conifer resin has existed within the genus for at least 35 million years (Rikkinen and Poinar 2000; Tuovila et al. 2013; Rikkinen et al. 2018; Rikkinen and Schmidt 2018). Recent estimates of divergence times of the Ascomycota place the separation of Mycocaliales and Eurotiomycetes in the Carboniferous (Prieto and Wedin 2013; Beimforde et al. 2014) and the origin of the Mycocaliciales crown group in the late Jurassic, when diverse conifer lineages were present (Lubna et al. 2021). It is possible that Mycocaliciales could have colonized conifers at an early stage of conifer evolution in the Permian, and it might well be that the resinicolous ecology evolved at a very early stage within Mycocaliciales. The oldest New Zealand pollen and macrofossil records of *Prumnopitys* and *Phyllocladus* are from Paleocene and Eocene deposits (Lee et al. 2016) and thus fungi on their exudates could have existed since then. Based on the isolated phylogenetic position of this clade from Podocarpaceae exudates, it could well be that this lineage diverged from other *Chaenothecopsis* clades in the Paleocene or even earlier.

## Supplementary Material

XML Treatment for Chaenothecopsis novae-zelandiae

XML Treatment for Chaenothecopsis matai

XML Treatment for Chaenothecopsis nodosa

## Supplementary materials

Supplementary material 1 Sampled specimens’ information for the three new *Chaenothecopsis* species from Podocarpaceae of New Zealand Data type table (word document) Explanation note Species name, collection/voucher number, collection date/sites, fungal hosts and locations. https://binary.pensoft.net/file/810920This dataset is made available under the Open Database License (http://opendatacommons.org/licenses/odbl/1.0/). The Open Database License (ODbL) is a license agreement intended to allow users to freely share, modify, and use this Dataset while maintaining this same freedom for others, provided that the original source and author(s) are credited.Christina Beimforde, Alexander R. Schmidt, Hanna Tuovila, Uwe Kaulfuss, Juliane Germer, William G. Lee, Jouko Rikkinen
