# Supplementary material for: ﻿Chaenothecopsis (Mycocaliciales, Ascomycota) from exudates of endemic New Zealand Podocarpaceae
Source: MycoKeys. 2023 Feb 16;95:101–29. doi: 10.3897/mycokeys.95.97601 (PMC10210246; doi:10.3897/mycokeys.95.97601)
Supplement: Supplementary material 1 — Sampled specimens’ information for the three new Chaenothecopsis species from Podocarpaceae of New Zealand [file mycokeys-95-101-s001.docx]

# **Table S1**. Sampled specimens’ information for the three new *Chaenothecopsis* species from Prodocarpaceae of New Zealand, including species name, collection/voucher number, collection date/sites, fungal hosts and locations. Specimens are deposited in the New Zealand Fungarium (PDD) Collection in Auckland.

| **Collection/Voucher number** | **Species name** | **Collection date** | **Host** | **Location** | **Degrees decimal minutes** |
| --- | --- | --- | --- | --- | --- |
| PDD110743/CBNZ001 | *Chaenothecopsis nodosa* | 25/02/2015 | *Prumnopitys taxifolia* | South Island, Dean Forest, approximately 20 km north of Tuatapere | 45°53,236 S,  167°38,7092 E |
| PDD110745/CBNZ039 | *Chaenothecopsis nodosa* | 5/04/2015 | *Prumnopitys taxifolia* | North Island, close to Pureora Forest, along Rimu walk close to Kakaho Camp site | 38°34,0224 S,  175°43,0525 E |
| PDD110744/CBNZ073B | *Chaenothecopsis novae-zelandiae* | 05/02/2017 | *Prumnopitys taxifolia* | South Island, Makarora | 44°13,787 S,  169°13,9708 E |
| PDD110742/JR13033 | *Chaenothecopsis novae-zelandiae/ Chaenothecopsis matai (mixed sample)* | 03/2013 | *Prumnopitys taxifolia* | North Island, close to Te Urewera, along Old State Highway 38 | 38°33,7592 S,  176°46,371 E |
| PDD110746/JR13032 | *Chaenothecopsis matai* | 02/3013 | *Prumnopitys taxifolia* | North Island, Te Urewera, along Ruatahuna Road | 38°35,5397 S,  176°49,3057 E |
| PDD110747/CBNZ037 | *Chaenothecopsis matai* | 04/2015 | *Prumnopitys taxifolia* | North Island, Pureora Forest, along Lagoon Walk | 38°38,9762 S,  175°40,0436 E |
| PDD110748/CBNZ08 | *Chaenothecopsis matai* | 03/2015 | *Prumnopitys taxifolia* | South Island, Otago, Taieri Mouth, along Bulls Creek walk | 46°3,2524 S,  170°11,1841 E |
| PDD110749/CBNZ070 | *Chaenothecopsis matai* | 17/02/2016 | *Prumnopitys taxifolia* | South Island, Croydon Bush, Dolamore Park | 46°3,6657 S,  168°49,9135 E |
